# Factors affecting expansion predictability of clear aligner treatment

**DOI:** 10.1007/s00784-025-06328-y

**Published:** 2025-04-21

**Authors:** Cristina de-la-Rosa-Gay, Eduard Valmaseda-Castellón, Rui Figueiredo, Octavi Camps-Font

**Affiliations:** 1 Department of Dentistry, Faculty of Medicine and Health Sciences, Universitat de Barcelona, Barcelona, Spain; 2 Group of Dental and Maxillofacial Pathology and Therapeutics, IDIBELL Research Institute, Barcelona, Spain; 3 Department of Dentistry, Facultat de Medicina i Ciències de la Salut, Universitat de Barcelona (UB), Campus de Bellvitge, Pavelló de Govern; 2a planta, Despatx 2.9 C/ Feixa Llarga s/n, L’Hospitalet de Llobregat, 08907 Spain

**Keywords:** Invisalign, Aligner, Aligner therapy, Aligner treatment, Clear aligner, Expansion, Orthodontic tooth movement

## Abstract

**Objectives:**

To determine the clinical factors associated with expansion predictability using clear aligners.

**Materials and methods:**

Pretreatment, prediction in the first approved ClinCheck, and pretreatment of the first refinement digital casts were recovered from Invisalign’s ClinCheck software for 98 patients with permanent dentition. Arch width measurements were collected in the ClinCheck arch width table for canines, first and second premolars, and first molars. Expansion predictability was calculated by subtracting the expansion achieved from that predicted. Expansion predictors were explored using univariate and multivariate generalized linear mixed models (GLMM).

**Results:**

Ninety-eight patients (mean age 48.7 years, standard deviation [SD] = 12.5 years) with 1440 eligible teeth (720 on each side) were assessed. The absolute difference between planned and achieved expansion was 0.92 mm (95% confidence interval [CI]: 0.86–0.99). While 72.2% of the measurements showed some degree of underexpansion, 79.3% of all overcorrections appeared in the mandible. According to the univariate analysis, the following variables were associated with expansion predictability: sex, arch, presence of posterior crossbite, absence of extractions, placement of attachments, absence of stripping, tooth type and higher predicted expansion. Those identified by GLMM were arch, tooth type, amount of predicted expansion and posterior crossbite.

**Conclusions:**

Expansion with Invisalign aligners is more reliable in the lower jaw and in the canine region. Cases with large, planned expansions or initial posterior crossbites (unilateral or bilateral) seem less predictable.

**Clinical relevance:**

The risk of not achieving the planned expansion is greater in the maxilla, posterior teeth, and when crossbite is present.

## Introduction

In recent years, clear aligners have become a useful and common tool for orthodontic treatments. These devices provide a more aesthetically pleasing and comfortable option than conventional fixed appliances, and are readily accepted by patients [1]. However, predictability is one of the major concerns of clinicians when prescribing clear aligners [2]. Several studies have calculated the predictability of different tooth movements, overbite correction and expansion with clear aligners [3–9]. Since transverse constrictions of the maxillary and mandibular arches are common alterations in daily practice, many studies have focused on the predictability of orthodontic aligners in achieving expansion. In the case of Invisalign polyurethane-based clear aligners, most authors have quantified the expansion obtained with Invisalign Exceed30 [10], now discontinued, while more recent reports have used SmartTrack [9, 11], which shows better adaptability and consistency of force transmission [12], despite not performing better in expansion [13]. Invisalign clear aligners are considered to be effective in achieving arch expansion, as they increase all the inter-tooth distances [14]. According to Tien et al. [9] and Houle et al., [15] more favorable outcomes are usually observed in the mandibular arch. Indeed, both of these studies found differences of over 10% between expansions in the upper and lower arches. Factors such as the number of attachments, the days of use, the need for interproximal reduction or dental extractions, could affect the expansion results. Other variables, such as the presence of posterior crossbite, implants or missing teeth, might also play an important role in the expansion achieved with aligners.

The accuracy of the prediction may vary. In general, it is greater in the premolar area [16, 17] and the mandibular arch [18], as reported by Tien et al. [9]. (76.4% on the maxillary arch and 86.9% on the mandibular arch) and by Houle et al. [15] (72.8% on the maxillary arch and 87.7% on the mandibular arch). However, the strict inclusion and exclusion criteria might limit extrapolation of the results to the general population wearing aligners, which is quite diverse. Thus, they only included patients changing aligners every 7 [9] or 14 [15] days, and without missing teeth [9, 15], bridges or dental implants [9], interproximal reductions performed in the premolar and molar area [9, 15], or the presence of third molars in the oral cavity [9]. These strict criteria strongly reduced the study group [9, 15]. Moreover, these reports did not adjust the results with factors that could affect the prediction of expansion, and mostly analyzed the area of expansion (canines or premolars versus molars) [16, 18]. Indeed, confounding effects and interactions should be explored by using multivariate models, in order to detect cases that are less likely to achieve the predicted expansion. Another important issue is that most studies perform multiple comparisons at different landmarks, but do not provide a multilevel analysis, which considers variation of both patient and tooth.

In addition, references exist not only to underexpansion [19, 20] but also to overexpansion [9, 15]. Thus, planning overexpansion with ClinCheck software (Align Technology, San José, CA, USA) to compensate for anticipated underexpansion, as other authors have suggested [14], might result in greater overexpansion and/or deficient occlusal contacts [13]. Regardless of whether overexpansion is incorporated to the treatment plans or not, it is still unclear which patients and areas are at most risk of underexpansion.

Thus, the aim of the present study was to determine how clinical variables affect the expansion predictability of Invisalign aligners in adult patients, and to build an explicative model of the expansion obtained, adjusted by these factors, using multi-level analysis.

## Materials and methods

A retrospective cohort study was conducted, comprising patients treated with SmartTrack version aligners (Align Technology, San José, CA, USA) from November 2017 to December 2023 by a single orthodontist (CRG) in a private dental clinic in the metropolitan area of Barcelona (Spain). This study design was selected due to its adequate cost-effectiveness relation since most variables were recorded consistently in the clinical appointments and could be assessed and measured objectively in a retrospective manner without compromising its reliability.

### Compliance with ethical standards

The present study was approved by the Ethics Committee of the Dental Hospital of the University of Barcelona (Barcelona, Spain) (Comitè d’Ètica i Investigació amb Medicaments de l’Hospital Universitat de Barcelona, protocol number: 2024-024-1), and was conducted in accordance with the Declaration of Helsinki [20]. The manuscript followed the Strengthening the Reporting of Observational Studies in Epidemiology (STROBE) guidelines [21]. Informed consent was not required, as the data was collected retrospectively and on an anonymized basis.

The following inclusion criteria were applied: (1) adult patients (> 18 years of age); (2) treatment completed with the aligners; (3) treatment not requiring dental extractions; and (4) treatment of both arches. The exclusion criteria were: (1) use of accessories other than attachments or class II / III elastics; (2) interruption of use of the aligners or poor compliance (misfits at most appointments); and (3) lack of a first refinement.

Diagnoses were reached through clinical examination, panoramic radiographs, teleradiography, intraoral and extraoral photographs, and stone or scanned casts. ClinChecks were reviewed by the orthodontist and corrected when necessary. Patients were treated with the Invisalign Comprehensive, Moderate or Lite versions.

The predictability of expansion was considered the primary outcome variable and was defined as the absolute difference (expressed in mm) between the expansion achieved (arch width after first stage treatment) and that planned (from the initial arch width) in canines, first and second premolars and first molars. Deviation calculations were performed using ClinCheck. Although the ClinCheck plan arch width table is reportedly measured at the point on the occlusal surface that corresponds to the long axis of a tooth, the algorithm and its validity have not been released by Align Technology [9]. 

The following data was also recorded for each patient: age at approval of the ClinCheck of the first stage of the Invisalign treatment, sex, presence of posterior crossbite (none, unilateral or bilateral, if at least one tooth from canine to second molar was in crossbite), Invisalign aligner version (Lite, Moderate, Comprehensive), number of planned aligners, treatment time from start of aligner use to first refinement, compliance (evaluated by the treating orthodontist based on the general fit of the aligners at the follow-up appointments: excellent if there were no misfits, and good in the case of occasional misfits), predicted expansion (mm), absolute discrepancy (mm), tooth extractions, predicted interproximal reduction in the upper and lower jaw (0 mm; ≤ 1 mm; 1–2 mm; > 2 mm), and number of attachments in the upper and lower jaw (0; 1 to 4; 5 to 8; ≥ 9). Misfits at most appointments was an exclusion criterion. Although the included patients did not undergo tooth extraction for orthodontics, some had missing teeth and/or dental implants before treatment. The number of implants in the upper and lower jaw, and the number of missing teeth in the upper and lower jaw (0; 1–2; > 2) were recorded. Patients were instructed to use the aligners for 7 or 10 days, according to the treatment modality (10 days for Lite and Moderate, and 7 days for the Comprehensive version). All data were retrieved by the treating orthodontist (CRG) from clinical records and the expansion table. Assessment of reliability was not needed, as the measurements were obtained from the Invisalign expansion table.

### Statistical analysis

The sample size was calculated using G*Power v.3.1.3 (Heinrich Heine University, Düsseldorf, Germany), based on the assumption that an absolute discrepancy of 0.5 mm would be clinically significant. Considering a common standard deviation (SD) of 0.83 mm [15], a risk of 0.05, and a statistical power of 80%, a total of 28 patients would be required. Since the teeth were not independent due to the three-level (patient, arch and tooth) data structure, the number of patients was adjusted. Assuming an intrasubject correlation of 0.5 (moderate), and that each subject had 2 arches and 8 assessed teeth, the final required sample size was 98 patients.

Data analyses were conducted using the IBM SPSS 29.0 statistical package for Windows (IBM Corp., Armonk, NY, USA). Simple generalized linear mixed models (GLMM) were generated to explore the homogeneity of scale and categorical variables. Three levels of analysis were taken into consideration: patient, arch and tooth. Crude mean differences (MD), with their respective 95% confidence intervals (95%CI), were calculated for each covariable.

A multivariate analysis was performed using a GLMM with a forced entry method. The dependent variable was the absolute difference between predicted and achieved expansion. All independent covariates with a p-value of less than 0.30 in the univariate analysis, as well as the interaction between arch and tooth, were included as predictors. Adjusted beta-coefficients and 95%CIs were obtained from the t-statistic, setting the level of significance at *p* < 0.05. The assumptions underlying the statistical analysis were checked.

## Results

The number of patients initially screened totaled 102, but 4 subjects were excluded due to treatment interruption (poor compliance). Thus, a total of 98 patients (720 analyzed tooth pairs) with a mean age of 42.25 years (SD = 12.5) were analyzed. Tables 1 and 2 show the main demographic and clinical variables of the study sample.

**Table 1:** Variables analyzed at patient and arch levels

	Category	*n* (%)	Mean (SD) ^a^	
Patient level (*n* = 98)				
Age (years)			42.32 (12.52)	
Sex	Male	29 (29.6)		
	Female	69 (70.4)		
Posterior crossbite	None	68 (69.4)		
	Unilateral	21 (21.4)		
	Bilateral	9 (9.2)		
Treatment modality	Lite	55 (56.1)		
	Moderate	6 (6.1)		
	Comprehensive	37 (37.8)		
Treatment time (months)			8.17 (11.41)	
Compliance ^b^	Good	4 (4.1)		
	Excellent	94 (95.9)		
Predicted expansion (mm)			2.46 (2.18)	
Absolute discrepancy (mm)			0.92 (0.84)	
Arch level (*n* = 196)				
Tooth extraction ^c^		1 (1.02) Maxilla 0 (0) Mandible		
No. of implants ^c^			0.15 (0.48) Maxilla 0.11 (0.45) Mandible	
No. of missing teeth			0.90 (1.26) Maxilla 0.82 (1.16) Mandible	
No. of attachments			7.67 (2.49) Maxilla 6.47 (2.13) Mandible	
Stripping (mm)			0.02 (0.18) Maxilla 0.20 (0.59) Mandible	
Predicted expansion (mm)			3.00 (2.12) Maxilla 1.92 (2.11) Mandible	
Absolute discrepancy (mm)			1.24 (0.93) Maxilla 0.61 (0.59) Mandible	

^a^ SD: standard deviation

^b^ Compliance was defined as the presence of misfits at follow-up appointments: excellent (no misfits) and good (occasional misfits). Poor compliance was an exclusion criterion

^c^ Tooth extraction and number of implants refer to the pre-treatment situation. Orthodontic treatment with extractions was an exclusion criterion

**Table 2:** Variables analyzed at tooth level (*n* = 720)

Tooth type	*n*	Predicted expansion (SD) ^a^	Achieved expansion (SD)	Absolute discrepancy (SD)
Maxilla				
Canine	98	2.26 (0.90)	1.41 (1.54)	0.90 (0.67)
First premolar	94	3.64 (2.13)	2.44 (1.78)	1.29 (0.93)
Second premolar	80	3.64 (2.22)	2.28 (1.61)	1.44 (0.95)
First molar	87	2.56 (2.01)	1.43 (1.22)	1.37 (1.07)
Mandible				
Canine	98	1.62 (1.74)	1.52 (1.54)	0.36 (0.32)
First premolar	97	2.21 (2.31)	1.89 (1.99)	0.69 (0.67)
Second premolar	91	2.35 (2.32)	2.11 (1.91)	0.67 (0.58)
First molar	75	1.43 (1.89)	1.45 (1.49)	0.78 (0.65)

^a^ SD: standard deviation

Accuracy analysis revealed an absolute overall difference of 0.92 mm between the predicted and obtained expansion (95%CI: 0.86 to 0.99). Signed discrepancies showed that 72.22% of the inter-canine, inter-premolar and inter-first-molar distances increased slightly less than predicted (95%CI: 68.84 to 75.37), while overcorrection was noted in 22.78% (95%CI: 19.86 to 25.98) of the cases. Notably, 130 of the 164 overcorrections occurred in the mandible and were distributed homogeneously among the different landmarks (*p* = 0.479).

The following variables were significantly associated with greater absolute discrepancy (*p* < 0.05) in the univariate analysis: the male sex, maxillary arch, presence of posterior crossbite, absence of extractions, placement of attachments, absence of stripping, posterior teeth, and higher predicted expansion (Table 3).

**Table 3:** Univariate generalized linear mixed models using absolute discrepancy (mm)

	Category	Coefficient (95%CI) ^a^	*P*-value
Age (years)		0 (-0.01 to 0.1)	0.577
Sex	Male	0	0.038
	Female	-0.23 (-0.44 to -0.13)	
Arch	Mandible	0	< 0.001
	Maxilla	0.59 (0.44 to 0.73)	
Posterior crossbite	None	0	< 0.001
	Unilateral	0.35 (0.15 to 0.54)	
	Bilateral	0.65 (0.25 to 1.04)	
Treatment modality	Lite	0	0.420
	Moderate	-0.28 (-0.61 to 0.15)	
	Comprehensive	-0.09 (-0.28 to 0.10)	
Compliance ^b^	Good	0	0.104
	Excellent	-0.53 (-1.17 to 0.11)	
Tooth extraction ^c^	No	0	< 0.001
	Yes	-0.17 (-0.26 to -0.07)	
No. of implants ^c^	0	0	0.933
	1	0.04 (-0.28 to 0.35)	
	≥ 2	-0.08 (-0.59 to 0.43)	
No. of missing teeth	0	0	0.343
	1 to 2	-0.02 (-0.23 to 0.18)	
	≥ 3	0.32 (-0.14 to 0.66)	
No. of attachments	0	0	< 0.001
	1 to 4	0.24 (-0.08 to 0.56)	
	5 to 8	0.46 (0.23 to 0.69)	
	≥ 9	0.80 (0.53 to 1.08)	
Stripping (mm)	0	0	< 0.001
	(0 to 1]	-0.43 (-0.78 to -0.07)	
	(1 to 2]	-0.34 (-0.52 to -0.15)	
	> 2	-0.44 (-0.74 to -0.15)	
Tooth type	Canine	0	< 0.001
	First premolar	0.36 (0.25 to 0.48)	
	Second premolar	0.44 (0.34 to 0.55)	
	First molar	0.46 (0.31 to 0.60)	
Predicted expansion (mm)		0.17 (0.12 to 0.21)	< 0.001
Treatment duration (months)		-0.03 (-0.07 to 0.01)	0.141

^a^ 95%CI: 95% confidence interval

^b^ Compliance was defined as the presence of misfits at follow-up appointments: excellent (no misfits) and good (occasional misfits). Poor compliance was an exclusion criterion

^c^ Tooth extraction and number of implants refer to the pre-treatment situation. Orthodontic treatment with extractions was an exclusion criterion

**Fig. 1:**
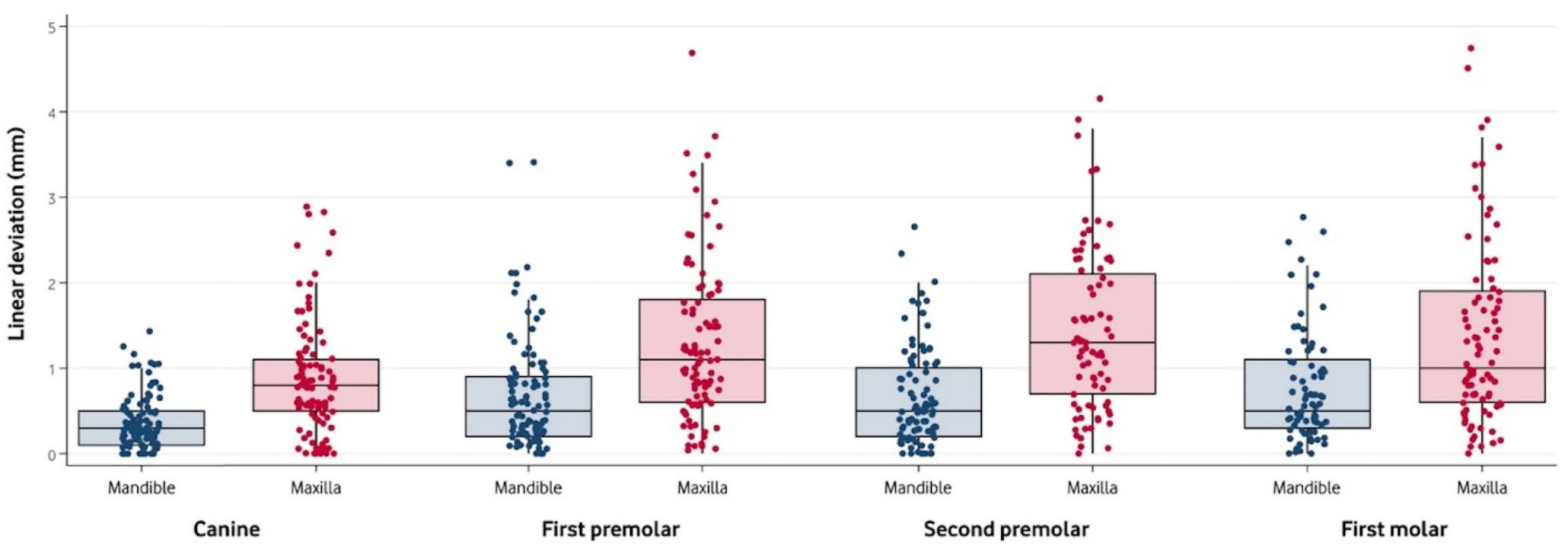
Boxplot of the absolute difference between predicted and achieved expansion by arch and tooth. Boxes indicate Q1, median and Q3. Single values are represented as scattered points. This image was created using STATA 14 (StataCorp LLC; College Station; TX, USA)

The final multivariate GLMM model included the following independent variables: arch, posterior crossbite, type of tooth, and predicted expansion. The effect of the interaction between arch and type of tooth was not significant (F[3, 709] = 1.43, p *=* 0.233). The results of the model are presented in Table 4. Figure 1 depicts the difference between predicted and achieved expansion by jaw and tooth.

**Table 4:** Multivariate generalized linear mixed model using absolute discrepancy (mm)

	Category	Coefficient (95%CI)^a^	*P*-value
Arch	Mandible	0	< 0.001
	Maxilla	0.47 (0.35 to 0.59)	
Posterior crossbite	None	0	< 0.001
	Unilateral	0.26 (0.08 to 0.45)	
	Bilateral	0.55 (0.20 to 0.90)	
Tooth type	Canine	0	< 0.001
	First premolar	0.22 (0.10 to 0.35)	
	Second premolar	0.29 (0.20 to 0.39)	
	First molar	0.45 (0.32 to 0.57)	
Predicted expansion (mm)		0.14 (0.10 to 0.19)	< 0.001

^a^ 95%CI: 95% confidence interval

## Discussion

A user-friendly tool to design the final position of the teeth and their occlusion is of great interest to orthodontists. The ClinCheck arch width table provides valuable information for correct diagnosis and treatment planning. This measurement tool seems to offer results similar to those of other methods such as simple software calipers [15], the MeshLab software measuring tool [9] or Meazure software [10, 22]. In addition, Solano-Mendoza et al. reported no significant differences between the initial ClinCheck and digital casts, with correct reproduction of the initial clinical situation in canine, first premolar, second premolar and molar gingival width; canine, first premolar and second premolar cuspid width; right and left molar rotation; molar inclination; and canine depth. However, they found some differences regarding molar cuspid width and arch depth (distance from the midpoint of the facial surface of the maxillary central incisors to a line connecting the cusps of the maxillary first molars) [10]. On the other hand, Meade et al. validated the use of Invisalign ClinCheck to measure overbite and overjet changes [23]. Measurements with reference points on occlusal surfaces, such as the ClinCheck plan arch width table, were more predictable than other methods using centroid or gingival margin reference points, such as MeshLab [9]. To the best of the authors’ knowledge, there is no evidence supporting that ClinCheck measurements are less reliable than other methods.

To the best of our knowledge, this is the first study to use a multilevel analysis for exploring the discrepancy between predicted expansion and that actually obtained with this system [17]. This is a key methodological issue: patients’ teeth are not independent, and analysis by tooth, despite its simplicity, might be inaccurate. In addition, the obtained expansion GLMM has been adjusted by factors that could be related to this discrepancy, thus reducing potential bias. In fact, 3 of these factors have proven to be relevant: dental arch, crossbite and tooth position. Specifically, discrepancies were higher in the upper arch, in patients with posterior crossbite, and in the molar area, which is consistent with other reports [16–18], despite differences in population or treatment planning. The fourth factor included was the amount of predicted expansion, which was positively correlated with discrepancy, i.e., a higher predicted expansion was less predictable. The use of multi-level analysis controlled for possible confounding factors by entering these variables in the final model.

The use of class II or class III intermaxillary elastics was not an exclusion criterion, in contrast to the use of intermaxillary elastics to potentiate the expansion of aligners (crossbite elastics). According to Bouchant et al., [17] the use of crossbite elastics should be considered a methodological bias.

As in other studies [9, 15, 22], in order to eliminate the effect of growth potential on the effectiveness of the aligners, only patients over 18 years of age were included. An analysis of expansion predictability in younger patients would require a different sample, focusing on different growth potentials.

The absolute difference between the planned and achieved expansion was 0.92 mm (95%CI: 0.86 to 0.99). While 72.2% of the measurements showed some degree of underexpansion, 79.3% of all overcorrections appeared in the mandible. Overcorrection was noted in 22.78% (95%CI: 19.86 to 25.98) of the total cases. Indeed, this overcorrection has been recommended in order to compensate for the low effectiveness of polyurethane-based aligners [13]. Tien et al. observed that overexpansion occurred for all teeth, excluding second molars. They considered that overexpansion could be related to planned bodily expansion, expressed as a greater tipping than planned [9]. 

In its early years, Invisalign recommended the use of each aligner for two weeks. At present, the duration has been reduced to one week. However, polyurethane-based aligners show cracks and roughness after use, though substance loss seems minimal [24]. Drake et al. compared these 2 times of use and concluded that material fatigue of Invisalign aligners does not play a significant role in tooth movement [25]. One could infer that 7 days are sufficient for the aligners to have their information expressed in tooth movement. However, we were unable to find studies comparing tooth movement results over different intervals of time. We also explored the effect of this variable (number of days) on predictability, since a one-week period could be riskier in non-fully compliant patients and in cases of complex malocclusions. In the present sample, the number of days for each aligner was decided according to the treatment modality involved (10 days for Lite and Moderate, and 7 days for the Comprehensive version). However, we found no relationship between expansion predictability and treatment duration or modality. An explanation for this outcome might be that the severity of malocclusion was probably a confounding factor, since it is associated with treatment results and modality.

Severe maxillary deficiencies that cause crossbites are more difficult to correct. Even with a conventional treatment approach using fixed appliances, they may require the additional forces provided by intermaxillary elastics or palatal fixed appliances, such as for instance a quad-helix [26]. In growing patients, orthopedic skeletal expansion may even be required [27, 28]. In adults, orthodontic movement is limited to tooth movement, and clear aligners tend to cause tipping; [17] thus, if more than 7 mm of expansion is required, surgically assisted rapid palatal expansion (SARPE [29]), mini-implant assisted maxillary expansion [30], or segmental osteotomies [31] may be required. Indeed, planning an overexpansion might not be the correct strategy to correct crossbite in adults, because it might result in overexpansion or excessive tipping, as our results and those of other reports suggest [9, 13, 14]. Thus, it is not surprising that expansion predictability was lower in the maxilla in patients with unilateral posterior crossbite, with even poorer results in cases with bilateral posterior crossbite, as shown in Table 4. Underexpansion, which is a common observation, is related to the existence of a transversal malocclusion [16, 17]. 

Attachments were introduced to increase force control and aligner retention. They can be placed automatically by the ClinCheck software or manually according to the criterion of the orthodontist. No differences in tooth movements have been found when comparing optimized and conventional attachments [7]. The univariate analysis showed that when the number of attachments increased, expansion prediction became less accurate. This might be related to the fact that the presence of posterior crossbite (unilateral or bilateral) was associated with a larger number of attachments. The number of attachments was not included in the multivariate model due to co-linearity.

With each aligner, clinicians obtain from 0.25 to 0.33 mm of tooth movement [6]. When greater expansion is required (due to greater arch compression), more aligners are needed and discrepancies become more likely. The present findings seem to support this statement, since large, planned expansions led to less accurate predictions. In this regard, Solano-Mendoza et al. found arch depth to be predictable in patients with molar expansions of under 2 mm [10]. 

In accordance with other studies [9], the results were more reliable in the lower arch. This is probably because aligners accomplish expansion with a tipping movement instead of a bodily translation movement of the teeth [22]. Compression of the lower arch is usually accompanied by lingual version of the teeth, which is easier to correct with aligners. This does not happen so often in the upper jaw: teeth in a compressed upper arch are frequently upright or even in buccal version. Thus, a bodily movement instead of mere tipping is desirable, although more difficult to achieve.

In the present study, expansion in the canine area turned out to be more reliable than in the premolar region. Indeed, expansion rates reportedly decrease progressively in the premolar and molar regions from mesial to distal [18]. In contrast, other studies reported expansion to be more predictable in premolars than canines [11, 13, 15]. A possible explanation is the restricted inclusion criteria of these studies, such as expansion of more than 3 mm or the absence of crossbite [9, 19],. Indeed, transverse deficiency and crossbite are more frequent in the premolar and molar areas than in canines, and predictability seems to be less reliable when greater expansions are needed or when crossbite are present.

The amount of interproximal reduction and the number of missing teeth were not significantly related to the predictability of expansion. Other retrospective studies found no differences in rotational and extrusive movements with Invisalign when using interproximal reduction or when spacing was present [7, 32]. Likewise, in the present sample, patient sex, age, the number of dental implants, treatment duration, or slight differences in compliance (good or excellent compliance) did not seem to be related to the predictability of expansion. Additionally, treatment duration is not only conditioned by the need for expansion but also by other movements, such as overbite correction or tooth rotations.

This study has some limitations that need to be considered. Firstly, its retrospective design might reduce the reliability of some variables. However, it is important to stress that most outcome variables were based on objective measurements with the use of specific software. Secondly, all the patients were treated by one experienced orthodontist with aligners from the same manufacturer, which might limit generalization of the results. Thus, further research is required to compare the different commercially available aligners systems. Another limitation is that the selection criteria were less restrictive than in previous reports, although this increased the potential for generalizing the results to the adult population using Invisalign, i.e., external validity. Finally, our study did not assess some factors that could affect the predictability of expansion, such as the type and complexity of the malocclusion, tipping, the periodontal support of each tooth, or the effect of simultaneous movements. Further research should explore these variables and relate them to the achievement of predicted expansion.

## Conclusions

Expansion with aligners is more reliable in the lower jaw and in the anterior area (canines). Cases with large, planned expansions and with initial posterior crossbites (unilateral or bilateral) seem to be less predictable.

## Data Availability

No datasets were generated or analysed during the current study.
